# Chemical disguise of myrmecophilous cockroaches and its implications for understanding nestmate recognition mechanisms in leaf-cutting ants

**DOI:** 10.1186/s12898-016-0089-5

**Published:** 2016-08-05

**Authors:** Volker Nehring, Francesca R. Dani, Luca Calamai, Stefano Turillazzi, Horst Bohn, Klaus-Dieter Klass, Patrizia d’Ettorre

**Affiliations:** 1 Centre for Social Evolution, University of Copenhagen, Copenhagen, Denmark; 2 Department for Ecology and Evolution, Biology I, Freiburg University, Hauptstr. 1, 79104 Freiburg, Germany; 3 Centro di Servizi di Spettrometria di Massa (CISM), University of Florence, Florence, Italy; 4 Dipartimento di Biologia, University of Florence, Florence, Italy; 5 Dipartimento di Scienza del Suolo e Nutrizione della Pianta, University of Florence, Florence, Italy; 6 Zoologische Staatssammlung München, Munich, Germany; 7 Senckenberg Naturhistorische Sammlungen Dresden, Dresden, Germany; 8 Laboratoire d’Ethologie Expérimentale et Comparée (LEEC), Université Paris 13, Sorbonne Paris Cité, Villetaneuse, France

**Keywords:** *Acromyrmex*, *Atta*, *Attaphila*, Camouflage, Cuticular hydrocarbons, Leaf-cutting ants, Mimicry, Myrmecophily, Nestmate recognition

## Abstract

**Background:**

Cockroaches of the genus *Attaphila* regularly occur in leaf-cutting ant colonies. The ants farm a fungus that the cockroaches also appear to feed on. Cockroaches disperse between colonies horizontally (via foraging trails) and vertically (attached to queens on their mating flights). We analysed the chemical strategies used by the cockroaches to integrate into colonies of *Atta colombica* and *Acromyrmex octospinosus.* Analysing cockroaches from nests of two host species further allowed us to test the hypothesis that nestmate recognition is based on an asymmetric mechanism. Specifically, we test the U-present nestmate recognition model, which assumes that detection of undesirable cues (non-nestmate specific substances) leads to strong rejection of the cue-bearers, while absence of desirable cues (nestmate-specific substances) does not necessarily trigger aggression.

**Results:**

We found that nests of *Atta* and *Acromyrmex* contained cockroaches of two different and not yet described *Attaphila* species. The cockroaches share the cuticular chemical substances of their specific host species and copy their host nest’s colony-specific cuticular profile. Indeed, the cockroaches are accepted by nestmate but attacked by non-nestmate ant workers. Cockroaches from *Acromyrmex* colonies bear a lower concentration of cuticular substances and are less likely to be attacked by non-nestmate ants than cockroaches from *Atta* colonies.

**Conclusions:**

Nest-specific recognition of *Attaphila* cockroaches by host workers in combination with nest-specific cuticular chemical profiles suggest that the cockroaches mimic their host’s recognition labels, either by synthesizing nest-specific substances or by substance transfer from ants. Our finding that the cockroach species with lower concentration of cuticular substances receives less aggression by both host species fully supports the U-present nestmate recognition model. Leaf-cutting ant nestmate recognition is thus asymmetric, responding more strongly to differences than to similarities.

**Electronic supplementary material:**

The online version of this article (doi:10.1186/s12898-016-0089-5) contains supplementary material, which is available to authorized users.

## Background

The coordination of complex societies requires precise communication. It is particularly important to defend the community from overt attacks and subtle forms of theft by outsiders. Potential intruders may be individuals from competing societies, but also specialised social parasites. The latter are a strong threat since they have evolved mechanisms to intrude efficiently.

Social insects use the most ancient modality to discriminate colony members from intruders: olfaction. Each individual bears a cuticular chemical profile, and between-colony variation in the profiles is informative about the colony identity, making the profile an ideal ‘nestmate recognition cue’ [1, 2]. When one individual (“discriminator”) encounters another individual whose cues do not match that of the discriminator’s colony, the discriminator typically attacks the encountered individual. The nestmate recognition process employed by social insects has led to adaptations by social parasites to copy their host’s chemical cues, either by synthesizing the respective substances and/or by acquiring them from their hosts [3, 4].

Nestmate recognition appears to be asymmetric. When individuals from two different colonies A and B meet, they are not necessarily equally aggressive towards each other, even if they are in principle equally motivated to attack intruders [5, 6]. The discrepancy between the cuticular profiles of colonies A and B may be perceived differently by A- and B-individuals. For instance, discriminators from colony A may perceive the odour dissimilarity as larger, and therefore be more likely to treat B-individuals as non-nestmates, than vice versa. This effect is possible because odour differences do not appear to be measured by the ants by a simple equivalent of Euclidean distance. Instead, it has been proposed that discriminators only react aggressively to non-nestmate profiles when these contain substances that are novel to the discriminator ants or when a given substance is more concentrated in the opponent’s cuticular profile than in the discriminator’s own profile (u-present model, [5, 7]). In the example laid out above, the odour blends of colonies A and B would include the same substances, but colony B would have an additional substance [5]. Such a recognition asymmetry can be caused by a desensitization of olfactory receptor neurons by the constant exposure to colony-specific substances. The neurons will then sensitively react to an increase in the quantity of any substance, but not to a reduction (pre-filter hypothesis [8]). The asymmetry could also be explained by a process of habituation, a form of non-associative learning [5]. In any case, social insects are more sensitive to differences than to similarities.

The “asymmetry hypotheses” also explain why callow workers can easily be transferred between social insect colonies without receiving aggression by non-nestmate workers. Callows bear a very low concentration of cuticular substances and will thus not be detected as intruders by ants with fully developed cuticular profiles [9]. Some social parasites exploit this effect to intrude host colonies by reducing the amount of recognition cues they bear (chemical insignificance, [4]). Lacking a profile can mean that the concentration of cuticular substances is generally low [10–13], or that the substances are not relevant for recognition (e.g. linear alkanes [14, 15]). Studying how social parasites gain entrance to social insect colonies can thus improve our understanding of the nestmate recognition process in general.

**Fig. 1:**
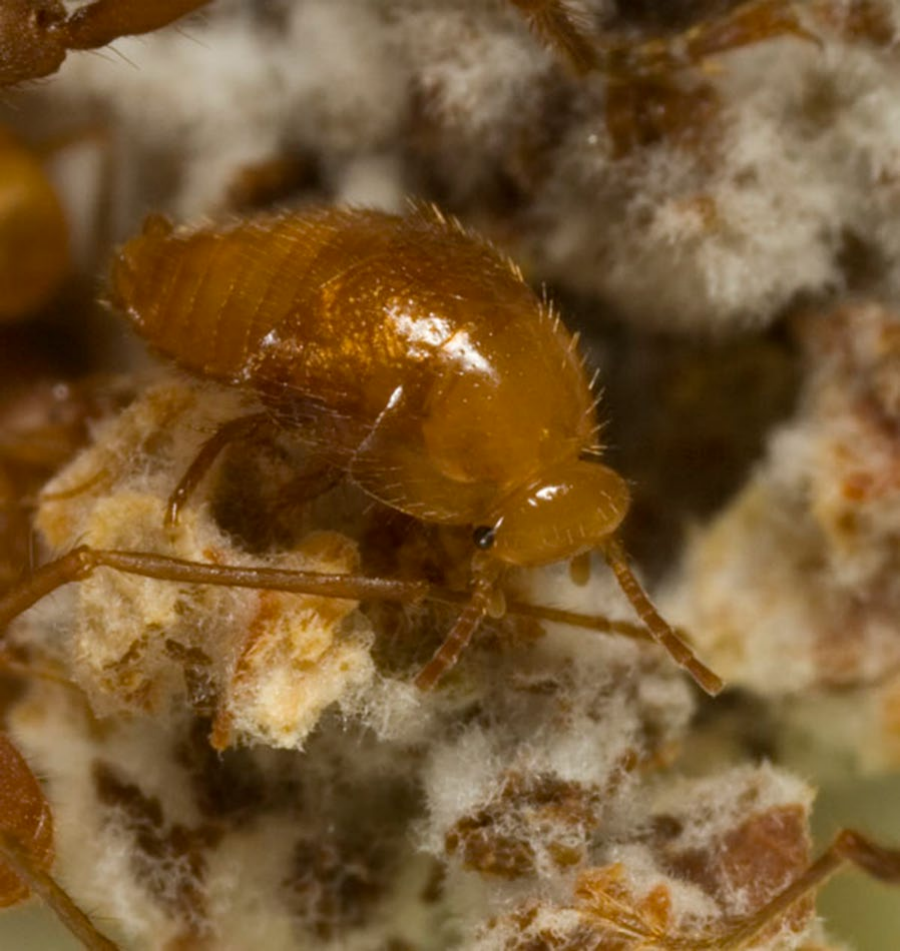
An *Attaphila* male on the fungus garden of a leaf-cutting ant colony

Using behavioural experiments and chemical analyses, we investigated the chemical strategies that myrmecophilous cockroaches of the genus *Attaphila* employ to enter leaf-cutting ant colonies. *Attaphila* are small (ca. 3 mm body length) cockroaches with apterous females and brachypterous males (Fig. 1). Leaf-cutting ant colonies are fruitful targets for social parasites because the ants cultivate a fungus with nutritional hyphae for food that intruders can also feed on. Hitherto, six different *Attaphila* species have been described, which are typically found in *Atta* and *Acromyrmex* colonies [16–21]. The cockroaches disperse between colonies by following the ants’ foraging trails and clinging to female *Atta* sexuals that depart for the nuptial flight [22, 23]. We collected *Attaphila* cockroaches from colonies of *Atta colombica* and *Acromyrmex octospinosus* leaf-cutting ants in Panama.

We tested whether the cockroaches bear colony-specific cuticular chemical profiles and whether cockroaches are recognised as intruders when they encounter discriminator ants from non-nestmate colonies. The experiments were designed to examine whether the cockroaches copy their host colony’s recognition label or evolved another way of intruding into host colonies. Having access to cockroaches from colonies of two different leaf-cutting ant genera that differ in the concentration of their cuticular chemical profile, we could also test the hypothesis that nestmate recognition is asymmetric.

## Results

### General observations

Ant workers from the two species were of similar size (U-Test n = 33, p = 0.81; head width *Atta* n = 18, $\bar{x}$ = 1.69 mm, sd = 0.31 mm; *Acromyrmex* n = 15, $\bar{x}$ = 1.71 mm, sd = 0.37 mm). Similarly, no morphometric measurements, including surface area, differed between *Atta*- and *Acromyrmex*-associated cockroaches (U-Tests, n = 42, p > 0.4 in all cases; head width of *Atta*-associated cockroaches n = 27, $\bar{x}$ = 1.52 mm, sd = 0.21 mm; *Acromyrmex*-associated cockroaches n = 15, $\bar{x}$ = 1.42 mm, sd = 0.35 mm). All cockroach morphometric measurements were highly correlated (typically r > 0.7, except eye distance with body and head length, where r = 0.66 and r = 0.50, respectively) and a principal component analysis yielded only a single principal component (PC) with an eigenvalue larger than one. Not surprisingly, nymphs were smaller than adults (generalized linear model (glm) on the PC, p = 0.04), but the ant species whose colonies the cockroaches were collected from did not affect the PC values (p = 0.55, interaction age x species p = 0.94).

Although *Attaphila* individuals from *Atta* and *Acromyrmex* colonies did not differ in size, closer inspection of their morphology revealed that they were in fact two different and so far not described species (HB, unpublished data). This was confirmed by sequences of the genes 12S, 16S, 18S, 28S, and H3 (Marie Djernæs and KK, unpublished data). The two species appear to be highly host-specific. All 24 *Atta*-associated cockroach individuals were categorized by morphology to belong to *Attaphila sp. A*, while all 11 *Attaphila* sp. *B* were associated with *Acromyrmex* colonies.

During the behavioural observations, several *Attaphila* individuals were observed to manipulate fungus fragments with their mouthparts, which suggests that they feed on the fungus and may thus negatively affect their host’s fitness, at least when occurring in large numbers.

### Behavioural experiment

**Fig. 2:**
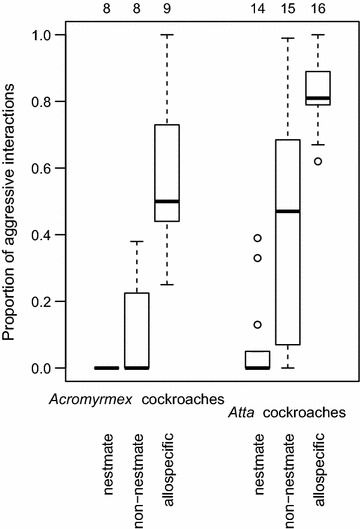
Aggression received by *Attaphila* cockroaches. Cockroaches associated with *Acromyrmex octospinosus* and *Atta colombica* colonies received aggression from non-nestmate, but hardly from nestmate workers. Allospecific workers were always more aggressive than conspecific non-nestmate workers, and cockroaches from *Atta* colonies received more aggression than those that were associated with *Acromyrmex* colonies. The *boxplots* indicate median (*horizontal mark*), interquartile range (*box*), data range, and outliers. The sample size is specified above the *boxes*

The aggression an *Acromyrmex*-associated cockroach received depended on the workers it encountered (glmm p < 0.001; Fig. 2); nestmate workers were not aggressive, while allospecific (i.e. *Atta*) workers were most aggressive. The aggression of conspecific (*Acromyrmex*) non-nestmates was intermediate and differed from that of nestmates (p = 0.036) and allospecifics (p = 0.005). The pattern was similar for *Atta*-associated cockroaches (interaction between cockroach and worker origin p > 0.99), but these received overall more aggression than *Acromyrmex*-associated cockroaches (factor cockroach origin p < 0.05; Fig. 2). In total, more cockroaches survived for 48 h in nestmate subcolonies (17 out of 22) than in non-nestmate and allospecific subcolonies (9/23 non-nestmate and 10/25 allospecific subcolonies, Pearson’s χ^2^ = 8.58, df = 2, p = 0.014;). Whether cockroaches would die could be predicted from the proportion of aggressive encounters, with a predicted 34 % of the cockroaches dying when there was no aggression, and 74 % dying when all encounters were aggressive (glm with binomial errors, n = 70, p = 0.015). Dead cockroaches were typically transported into the subcolony’s trash pile. Mortality among non-attacked cockroaches was likely due to the aggression test arenas not being optimal for long-term housing (cockroaches did not find suitable hideouts and were constantly fleeing from ants, and may have suffered from desiccation).

### Chemical analysis

**Fig. 3:**
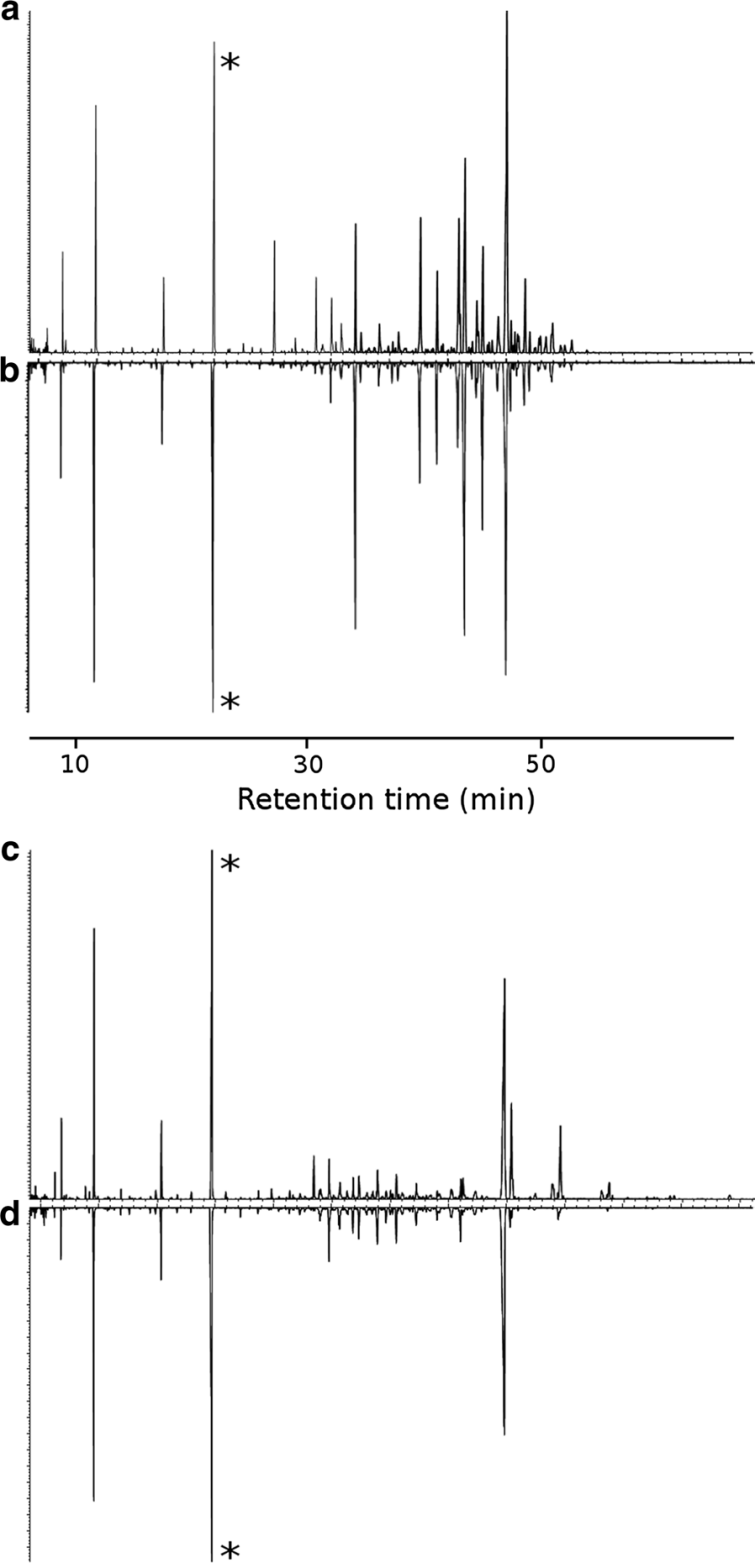
Cuticular chemical profiles (gas chromatograms) of cockroaches and leaf-cutting ant workers. **a** *Atta* worker; **b** *Atta*-associated cockroach; **c** *Acromyrmex* worker; **d** *Acromyrmex*-associated cockroach. The profiles of the cockroaches are more similar to those of their host workers than among each other. All profiles include the peak of an internal standard (*)

We found qualitative differences between samples collected from nests of the two different ant species, but no qualitative differences between cockroaches and workers collected from nests of the same species (Fig. 3; Table 1). In total, 46 GC-peaks of *Acromyrmex* workers and *Acromyrmex*-associated cockroaches, and 44 peaks from *Atta* workers and *Atta*-associated cockroaches were used for further analysis (Table 1). The chemical profiles of *Acromyrmex* workers and *Acromyrmex*-associated cockroaches consisted largely of a row of unsaturated hydrocarbons that were absent from *Atta* colonies (C29:1, C31:1, C31:2, C31:1, C37:2; Table 1). Specific to samples from *Atta*-colonies were trimethyls C31 and C34, which were very abundant in these samples. Samples from colonies of both species contained large quantities of docoseneamide and small amounts of octadecenamide. Amides have been previously found in nests of leaf-cutting ants by Richard et al. [24], who suggested that they may be produced by the symbiotic fungus. Such substances have otherwise rarely been described from social insects, apart from the Dufour gland and inside of the body of *Polistes* paper wasps [25]. These unusual substances could in theory be contaminants from plasticware, but are also known to be used in arthropod communication [26, 27].

**Table 1:** The cuticular substances of *Acromyrmex octospinosus* and *Atta colombica* workers and *Attaphila* cockroaches

No.	Substance	Rt	Type	*Acromyrmex* colonies				*Atta* colonies			
				Worker		Cockroach		Worker		Cockroach	
				$\bar{x}$	sd	$\bar{x}$	sd	$\bar{x}$	sd	$\bar{x}$	sd
1	Unidentified	5.3	Other	0.34	0.23	0.70	0.73	0.32	0.30	0.77	1.11
2	C12:OH	6.7	Other	2.42	1.46	2.86	2.00	2.48	2.10	5.21	2.23
3	n-C18	11.2	Linear	0.64	1.36	1.14	1.20	0.06	0.07	0.27	0.60
4	C16-OH	12.6	Other	0.21	0.21	0.30	0.28	–	–	–	–
5	n-C20	14.9	Linear	0.06	0.05	0.83	0.96	–	–	–	–
6	C18-OH	16.6	Other	0.38	0.22	0.61	0.26	0.27	0.40	0.35	0.27
7	n-C22	19.0	Linear	0.10	0.07	0.64	0.70	0.03	0.02	2.19	8.89
8	n-C23	20.9	Linear	0.31	0.24	0.29	0.29	0.26	0.12	0.15	0.19
9	Octadecenamide	22.1	Other	0.70	0.53	0.46	0.49	0.44	0.61	0.32	0.42
10	n-C24	22.8	Linear	0.24	0.17	0.77	0.79	0.26	0.16	0.26	0.50
11	n-C25	24.8	Linear	1.19	0.79	0.55	0.53	9.07	2.84	0.36	0.36
12	n-C26	26.6	Linear	0.52	0.22	0.78	0.83	0.86	0.26	0.41	0.50
13	7-, 8-, 9-, 10-, 11-, 12-, 13-MeC26	27.2	Methyl	1.01	0.55	1.06	0.48	0.61	0.27	0.99	0.36
14	n-C27	28.4	Linear	4.55	2.40	0.59	0.44	4.83	1.11	0.53	0.33
15	7-, 9-, 11-, 13-MeC27	28.9	Methyl	1.94	1.14	2.33	1.53	0.83	0.57	1.71	0.92
16	Docosenamide	29.7	Other	13.97	14.65	15.57	11.07	8.37	5.93	8.86	8.16
17	n-C28	30.0	Linear	0.55	0.39	0.67	0.68	0.68	0.33	1.51	3.83
18	9-, 10-, 11-, 12-, 13-, 14-MeC28	30.6	Methyl	2.87	4.27	3.47	6.17	1.07	0.71	1.95	1.21
19	C29:2a	30.7	Unsat	0.45	0.68	0.19	0.19	–	–	–	–
20	C29:2b	30.8	Unsat	0.47	0.62	0.27	0.24	–	–	–	–
21	C29:1	31.4	Unsat	10.90	12.01	2.60	2.31	–	–	–	–
22	n-C29	31.8	Linear	3.06	1.92	5.61	1.82	9.93	3.83	12.19	6.00
23	9-, 11-, 13-MeC29	32.2	Methyl	3.88	2.53	4.64	2.21	1.41	0.88	3.05	2.38
24	3-MeC29 + methylated alkanes	32.9	Methyl	1.32	1.22	0.95	0.69	3.09	3.95	2.18	2.99
25	10-, 11-, 12-, 13-, 14-, 15-MeC30	33.8	Methyl	3.60	2.87	2.35	1.30	2.19	1.47	3.91	2.61
26	C31:2	34.1	Unsat	5.88	9.33	5.94	3.61	–	–	–	–
27	C31:1	34.6	Unsat	3.71	3.81	5.65	2.50	–	–	–	–
28	N-C31 and 2Me-C30	34.9	Linear	1.31	0.87	4.06	2.88	0.81	0.49	1.49	0.81
29	9-, 11-, 13-, 15-Me-C31	35.4	Methyl	4.26	2.44	5.18	1.80	1.44	1.04	2.52	1.61
30	7,11-diMe-C31	36.0	Methyl	–	–	–	–	0.55	0.81	0.84	0.86
31	10-, 11-, 12-, 13-, 14-, 15-, 16-MeC32	37.2	Methyl	1.62	1.86	2.19	0.97	–	–	–	–
32	3,7,11-triMe-C31	37.3	Methyl	–	–	–	–	11.91	3.17	7.95	3.44
33	C33:2	37.4	Unsat	4.02	4.49	6.83	3.37	0.22	0.09	0.22	0.16
34	8,12-diMe-C32	37.7	Methyle	0.84	0.90	1.33	1.31	–	–	–	–
35	C33:2	37.7	Unsat	0.84	0.90	1.33	1.31	0.98	2.86	0.69	2.37
36	6,10-diMe-C32	37.9	Methyl	–	–	–	–	0.15	0.13	0.33	0.25
37	n-C33 + methylated alkane	38.2	Linear	0.71	0.52	0.93	0.43	0.27	0.22	0.84	0.61
38	9-, 11-, 13-, 15-MeC33	38.8	Methyl	2.04	1.73	2.80	1.21	–	–	–	–
39	9,13-diMe-C33	39.5	Methyl	0.59	0.69	0.14	0.11	0.35	0.68	0.18	0.11
40	3Me-C33	40.0	Methyl	1.49	1.43	1.27	1.51	0.37	0.31	1.28	1.77
41	C35:1 + C35:2	41.0	Unsat	3.33	4.31	5.31	4.43	–	–	–	–
42	Methyle alkane mixture	42.0	Methyl	–	–	–	–	4.74	1.63	4.69	1.52
43	4,8,12-triMe-C34	42.6	Methyl	–	–	–	–	12.60	4.34	14.60	5.91
44	13-, 15-, 17-MeC35	42.6	Methyl	0.64	0.78	1.11	0.40	–	–	–	–
45	x-MeC36:1	42.9	Unsat	–	–	–	–	0.40	0.47	0.35	0.75
46	x-MeC36:1	43.1	Unsat	–	–	–	–	0.31	0.25	0.91	3.06
47	x-MeC36:1	43.3	Unsat	–	–	–	–	0.46	0.28	0.32	0.21
48	5,9- and 5,11-diMe-C35	43.9	Methyl	–	–	–	–	3.75	1.21	3.32	1.09
49	3,7,11-triMe-C35	45.0	Methyl	–	–	–	–	5.05	6.62	3.81	2.00
50	C37:2 + C37:1	45.0	Unsat	5.59	6.78	3.50	2.07	–	–	–	–
51	x-MeC37:1	45.3	Unsat	–	–	–	–	0.55	0.72	0.65	0.83
52	4,8,12-triMe-C36	46.6	Methyl	–	–	–	–	2.61	0.79	2.65	0.77
53	x-MeC38:1	47.0	Unsat	0.75	0.42	0.64	0.54	1.07	0.72	0.93	0.70
54	x-MeC40:1	48.5	Unsat	–	–	–	–	2.16	1.23	2.47	1.22
55	x-MeC40:1	49.2	Unsat	4.44	5.83	1.96	1.25	0.66	0.30	0.59	0.27
56	x-MeC41:1	50.1	Unsat	–	–	–	–	1.13	0.43	0.99	0.35
57	x-MeC41:1	51.4	Unsat	–	–	–	–	0.37	0.47	0.22	0.11
58	x-MeC41:2	53.8	Unsat	0.72	1.03	0.21	0.20	–	–	–	–
59	13-, 15-Me-C41	55.1	Methyl	0.38	0.47	0.13	0.10	–	–	–	–
60	Alkyl ester 1	58.8	Other	0.55	0.70	0.20	0.29	–	–	–	–
61	x-MeC44:1	59.2	Unsat	0.53	0.50	0.30	0.19	–	–	–	–
62	Alkyl ester 2	63.9	Other	0.90	0.83	0.15	0.09	–	–	–	–

Cockroach samples are separated according to the ant species they were collected with. Shown are mean and standard deviations of the relative abundances as well as the retention time (Rt) and substance class (Type). Some substances were not found in one of the species (−)

While there were no qualitative differences, short-chained *n*-alkanes (*n*-C20 up to *n*-C22) were more abundant in the *Acromyrmex*-associated cockroaches than in *Acromyrmex* ants. In contrast, longer-chain *n*-alkanes (mainly *n*-C27) were more abundant in *Acromyrmex* workers (Table 1). Overall, however, the relative amount of linear alkanes did not differ between workers and cockroaches (ANOVA p = 0.087, and neither did we find differences for methylated, unsaturated, or non-hydrocarbon substances (ANOVA p > 0.35 in all cases; Table 2).

**Table 2:** Percentage of different substance classes from the total cuticular chemical profile of ants and cockroaches

Substance class	*Acromyrmex* cockroaches	*Acromyrmex* workers	*Atta* cockroaches	*Atta* workers
Linear Alkanes	16.9 ± 6.4	13.2 ± 5.3	20.2 ± 8.5	27.1 ± 6
Branched Alkanes	29.6 ± 9.6	26.8 ± 12.8	56.7 ± 12.4	53.1 ± 6.7
(Branched) Alka(di)enes	34.3 ± 10.6	41.7 ± 19.1	8.3 ± 4.4	8.3 ± 3.2
Non-Hydrocarbons	20.1 ± 10.6	18.2 ± 14.7	14.7 ± 7.7	11.6 ± 6.3

Mean ± standard deviation

**Fig. 4:**
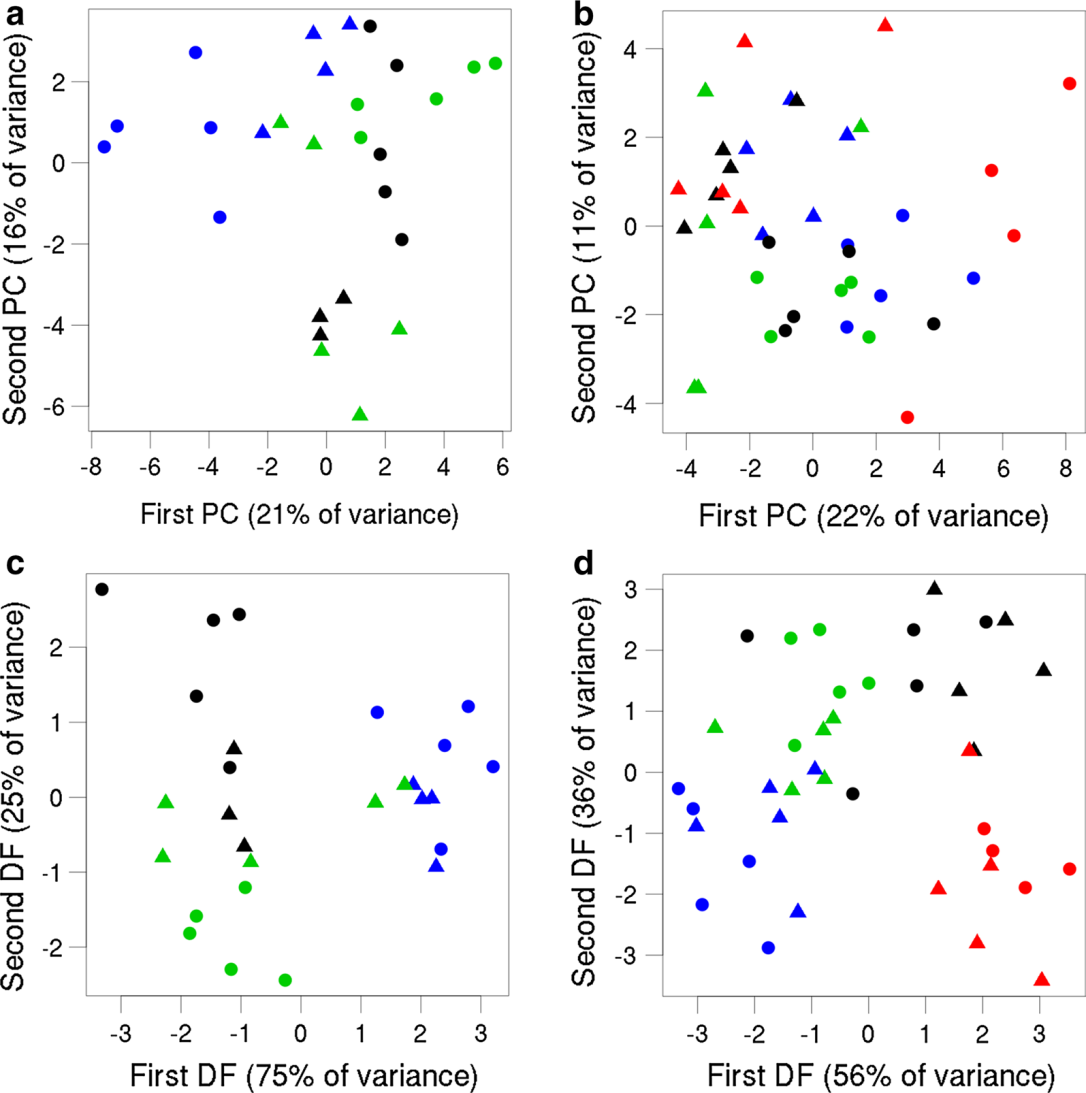
Multivariate representations of cockroach and worker cuticular chemical profiles. The first two principle components (PCs, *panels* **a**, **b**) and discriminant functions (DFs, *panels* **c**, **d**) for chemical profiles of workers and cockroaches from *Acromyrmex* (**a**, **c**) and *Atta* (**b**, **d**) colonies. The discriminant analyses were set up to discriminate between colonies. *Triangles* represent cockroaches and *circles* workers; colours code for the different colonies

The difference between *Acromyrmex* workers and *Acromyrmex*-associated cockroaches was also evident in a multivariate analysis (Fig. 4a; Wilks MANOVA using the first four principal components (PCs), n_W_ = 15, n_C_ = 12, λ = 0.21, p < 0.001), and profiles also differed between colonies (λ = 0.26, p < 0.001). We did not find a difference between the profiles of cockroach adults (only n = 3) and nymphs (n = 9, λ = 0.89, p = 0.53). The difference between *Acromyrmex* workers and *Acromyrmex*-associated cockroaches was evident in the second, third, and forth PC, where the linear alkanes described above had high loadings (Additional file 1). In a discriminant analysis, 22 out of 27 *Acromyrmex* samples (n_C_ = 12, n_W_ = 15) were correctly classified according to their colony (81 %), which is significantly more than would be expected by chance (Fig. 4c; p < 0.001, as compared to a median of 48 % , 95 %-quantile of 63 %, and maximum of 74 % in a permutation test with random groups); all five misclassified samples were cockroaches.

*Atta* workers and *Atta*-associated cockroaches did not differ in the relative abundance of branched or unsaturated alkanes and non-hydrocarbons (ANOVA p > 0.09 in all cases; Table 2); however, workers had higher relative amounts of linear alkanes (p = 0.02), which was mainly caused by differences in *n*-C25 and *n*-C27 (Table 1). In the multivariate analysis, the cuticular profiles of samples collected from *Atta* colonies also differed between cockroaches and workers (Fig. 4b; Wilks MANOVA, n_W_ = 19, n_C_ = 20, λ = 0.14, p < 0.001) and between colonies (λ = 0.03, p < 0.001) in a MANOVA on the first eight principal components. There may be a difference between cockroach life stages (6 adults and 14 nymphs; λ = 0.61, p = 0.07). Inspecting the contribution of the eight PCs and the loadings of all substances on each PC separately revealed that the difference between workers and cockroaches was mostly evident in the two first PCs (see Fig. 4b; Additional file 1), but no single substances were responsible for this difference as loadings were evenly distributed on these PCs. The discriminant analysis correctly identified the colony origin of 34 out of 39 samples (n_C_ = 20, n_W_ = 19) from *Atta* colonies (87 %; in a permutation test with random grouping, a median of 38 %, 95 %-quantile of 49 %, and maximum of 59 % of the samples were classified correctly; p < 0.001; Fig. 4d). The algorithm predicted the colony for three workers and two cockroaches incorrectly.

**Fig. 5:**
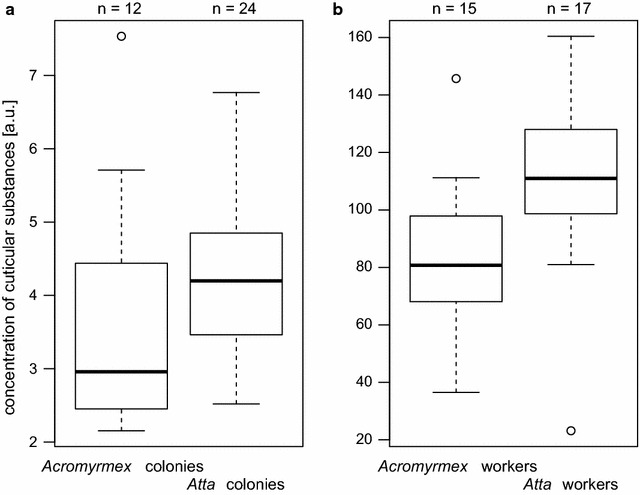
The concentration of all cuticular substances for *Attaphila* cockroaches (**a**) and for ant workers (**b**). The units are arbitrary since the concentrations per surface area could not be calculated exactly. The measurements only serve to compare among ants and cockroaches, respectively. Values are not comparable between ants and cockroaches. *Boxplots* indicate median (*horizontal mark*), interquartile range (*box*), data range, and outliers; sample size is specified above the boxes

The concentration of cuticular substances on workers from *Acromyrmex* colonies was lower than on those from *Atta* colonies (U-Test, n = 32, p < 0.01; Fig. 5), and there was a similar trend in the cockroaches (n = 36, p = 0.067).

## Discussion

*Attaphila* cockroaches were mostly attacked by non-nestmate but rarely by nestmate workers, suggesting that the cockroaches bear colony-specific recognition cues that can be detected by the ants. Our chemical analyses confirmed this: the cockroaches did not only share a species-specific cuticular chemical profile with their host ants, but also a colony-specific label, which is often found to be the case in social parasites and myrmecophiles [4, 28–30]. The cockroaches could blend into their host colonies either by acquiring a colony-specific chemical profile through substance transfer (camouflage [4]) or by synthesizing relevant chemicals (mimicry [31]). Currently we cannot exclude either possibility. As the cockroaches from *Atta* and *Acromyrmex* appear to be two specialised species, mimicry would be a reasonable explanation. Alternatively, both cockroach species could bear a similar “totipotent” profile that can be camouflaged into resembling any ant species by substance transfer. To discern these two hypotheses, cockroaches would need to be “crossfostered” for a while in colonies of another host species (e.g. *Atta*-associated cockroaches crossfostered in *Acromyrmex* colonies) or be kept in isolation before the cuticular chemical profiles are sampled.

Hypotheses regarding the other chemical mechanism for disguise, chemical insignificance, are comparably hard to test in the *Attaphila*-attine system. Ant and cockroach morphology differ vastly, so that it is difficult to calculate an estimate of surface area that is reliably comparable between ants and cockroaches. We can thus not test whether the cockroach cuticular chemical substances are less concentrated than those on the ants, which would be one way to achieve insignificance. The other way would be a relative reduction of recognition-relevant substances within the chemical profile, with no need for systematic variation in the total substance concentration. *Acromyrmex insinuator*, an ant social parasite of *Acromyrmex echinatior*, reduced the overall concentration of the cuticular hydrocarbons and in addition produces relatively large quantities of linear alkanes as compared to its host [29, 32]. Linear alkanes are often not used for nestmate recognition, thus their overproduction may be a way to achieve chemical insignificance [7, 15]. Indeed, the chemical profiles differed between cockroaches and ant workers. However, we did not find any general pattern in the relative abundance of different substance classes. One exception is that *Atta* workers bear more *n*-alkanes than *Atta*-associated cockroaches, so that there is currently no evidence for chemical insignificance in the cockroaches. That said, leaf-cutting ants may be a special case with regard to the substances used for nestmate recognition. Non-hydrocarbons, in particular volatile substances, appear to be involved in leaf-cutting ant nestmate recognition [33, 34]. The overall picture is not entirely clear since the substances in question vary between species and non-volatile substances and hydrocarbons such as those we analysed in this paper also play a role (VN, unpublished data, [35–37]). In any case, our analysis may have missed some very volatile substances from the ants’ glands that could potentially also affect nestmate recognition, and a formal analysis of the substances used in different leaf-cutting ant species would be in order before drawing any final conclusions regarding chemical insignificance in *Attaphila* cockroaches.

Chemical strategies may not be the only possibility to facilitate social parasitism. During the behavioural experiments, the cockroaches seemed to actively avoid inspection by ant workers. Cockroaches also often hid in crevices in the fungus. This behaviour exposed only their round and smooth backs (Fig. 1; cf. [16]), making it difficult for ants to grab or bite the cockroaches. Successful attacks by the ants seem unlikely unless a cockroaches becomes exposed, for example when it is flipped onto its back.

We found that the concentration of chemical substances on workers and cockroaches collected from *Atta* colonies was higher than that on workers and cockroaches from *Acromyrmex* colonies. The difference allows for a correlative test of the hypothesis that nestmate recognition is asymmetric, i.e. sensitive to increases but ignorant to a reduction of profile concentration [5, 8]. All else being equal, individuals with lower concentrations of recognition cues should be less likely to be attacked by discriminators [13]. Even if relative abundance of substances in the recognition labels differed between the discriminator and a non-nestmate, the discriminator is expected to not perceive these differences if the substances in question are below a hypothetical physiological detection threshold (low concentration, [38]). Therefore, theory predicts that cockroaches from *Acromyrmex* colonies should receive less aggression than those from *Atta* colonies. Indeed, we observed this pattern in our aggression experiments with cockroaches from nests of the two leaf-cutting ant species. Our results fully support the U-present model [5] and are compatible with the pre-filter hypothesis [8]. Further experiments, including experimentally manipulated cuticular profiles and electrophysiology, specifically tailored to test the models, are called for to refine our understanding of ant nestmate recognition.

## Conclusions

*Attaphila* cockroaches found in colonies of *Atta colombica* and *Acromyrmex octospinosus* bear colony-specific recognition cues. Thus, the cockroaches are accepted by nestmate but attacked by non-nestmate ant workers. A comparative analysis of chemical and behavioural data supports that nestmate recognition is based on an asymmetric mechanism, i.e. it is more sensitive to an increase than to a reduction of chemical recognition cues.

## Methods

### Animals

We collected fragments of six mature *Atta colombica* ant colonies and four entire *Acromyrmex octospinosus* ant colonies that contained individuals of *Attaphila* cockroach species in Gamboa, Panama. We set up the colonies in plastic bowls (20–30 cm diameter), with Fluon-covered sides, in a laboratory close to the field site. The fungus gardens of the ant colonies were covered by smaller plastic bowls (10–20 cm diameter) to keep the appropriate humidity. The colonies were housed under natural temperature and light conditions and were fed with *Lagestroemia speciosa* leaves and mango fruits. We used cockroaches and ant workers in behavioural experiments and also analysed their cuticular chemicals.

### Behavioural experiment

We set up subcolonies with 200 mg of fungus in Petri dishes (6 cm diameter), the minor workers that were naturally present in it, four medium workers with a head width of 1–2 mm, and one larger worker (2–2.5 mm head width). The lid was closed during all experiments, and half of the dish’s floor was covered with moist filter paper to prevent desiccation. We let the ants acclimate for 1 h, and then introduced a single cockroach through a small hole in the lid. We observed the behaviour of the ant workers towards the cockroach for 5 min and counted the aggressive (threat, bite) and non-aggressive (antennation, indifference) interactions.

We used 45 cockroaches from the six *Atta colombica* “donor” colonies and 25 cockroaches from the four *Acromyrmex octospinosus* donor colonies for the aggression tests. Each donor colony was paired with a non-nestmate colony of the same ant species and an allospecific colony (*A. octospinosus* colonies for *Atta*-associated cockroaches and vice versa) from which subcolonies were prepared. Nestmate subcolonies contained workers and fungus from the donor colony itself. For the aggression tests, roughly equal numbers of cockroaches were introduced into nestmate, conspecific non-nestmate, and allospecific subcolonies. All subcolonies and cockroaches were used in one aggression test only, and the experimenter was blind to the origin of the cockroaches. After the behavioural observations, we kept the subcolonies intact for 48 h and regularly checked whether the cockroaches were still alive.

For each aggression test, we calculated the proportion of encounters that were aggressive and analysed them in a generalized linear mixed model with binomial errors. We used the ant species the cockroach was associated with (*Atta* vs *Acromyrmex*) and the origin of the ant workers relative to the cockroach (nestmate, non-nestmate, or allospecific colony) as fixed factors, and the identity of the colony the cockroach was collected from as a random factor. We tested for the significance of the fixed factors and their interaction using log likelihood and Akaike information criteron [39].

### Chemical analysis

We extracted the cuticular chemical profiles of 3–5 cockroaches and 4–5 medium sized workers from each of three *Acromyrmex octospinosus* and four *Atta colombica* colonies by immersing each freeze-killed individual in 200 µl of *n*-pentane for 5 min. We did not extract individuals from all ten colonies used in the behavioural experiment because some of these did not contain sufficient numbers of cockroaches. The pentane was evaporated and then 15 µl of *n*-heptane were added, which contained 5 ng/µl of nonadecanoic acid as internal standard. Three microlitres of the sample were then injected into an Agilent 7890A gas chromatograph (ZB-5 column 30 m × 0.25 mm, 0.1 µm thickness) coupled to a 5975C mass spectrometer. The column temperature was initially held at 70 °C for 1 min, then increased by 30 °C/min to 150 °C, then by 4 °C/min to 270 °C, at 2 °C/min to 310 °C, and finally at 4 °C/min to 320 °C, where it was held for 10 min. Inlet and transfer line were set to 300 °C.

We integrated the areas under peaks that contributed at least an average of 0.1 % to the total chemical profile of all samples of an ant species or cockroaches from colonies of an ant species. We transformed the peak areas of all substances but the internal standard according to Aitchison [40] and submitted the transformed areas to two separate principal component analyses [41], one for all samples collected in *Acromyrmex* colonies, and the other for all samples from *Atta* colonies. We used the principal components (PCs) judged informative by the broken stick method [42] for a MANOVA with the factors species (cockroach vs. ant worker), cockroach life stage (adult or nymph, based on presence/absence of tegmina and the morphology of the terminal sternites), and colony identity. We also exploratorily checked which principal components contributed to any effects found in the MANOVA and inspected the factor loadings for conspicuous substance variation. To investigate whether the cuticular profiles of cockroaches were as colony-specific as those of ant workers, we conducted a discriminant analysis with leaving-one-out crossvalidation using colony identity as the only grouping variable, not differentiating between ants and cockroaches [43]. We estimated p-values for the discriminant analysis in a permutation test with 1000 randomly drawn groups [44].

We calculated the relative amounts of different substance classes (linear alkanes, branched alkanes, unsaturated hydrocarbons, other substances) and compared them (log-transformed) between cockroaches and ant workers and among colonies using ANOVA. In samples from *Atta* colonies, two linear alkanes co-eluted with methyl-alkanes (Table 1). These two substances could not be separated for the analysis. We thus conducted the analysis twice, once attributing the peaks as linear alkanes and once as methyl-alkanes. The results obtained were not qualitatively affected and in this manuscript we report the results for classifying the peaks as linear alkanes.

We estimated the variation in cuticular substance amounts across individuals from the internal standard’s area relative to that of all other substances. Since the substance amount per individual is likely to depend on the individual’s size and surface area, we also took morphometric measures of the samples used in the chemical analysis. After extracting the cuticular substances, we transferred the samples individually into vials with ethanol for storage until we could take the morphometric measurements. Four cockroach and two worker samples lack morphometric measurements since labels on ethanol samples were lost during transport; these individuals were omitted from the analysis of substance concentrations. We measured head width for ant workers and body and head length and width, as well as minimum eye distance, for cockroaches. We approximated cockroach surface area treating the cockroach as a prolate spheroid with

$$A = 2\pi a^{2} \times \left( {1 + \frac{c}{a \times e} \times \arcsin (e)} \right)$$

where a and c are body width and length and $e = \sqrt {\frac{{1 - a^{2} }}{{c^{2} }}}$. As we cannot estimate worker body size by head width alone, we used squared head width as a proxy since surface scales quadratically with diameter and related measures (however, using linear or squared head width made no difference in the effects observed). We then tested whether the substance concentration, i.e. amount per surface area, differed between samples from *Acromyrmex* and *Atta* colonies. Note that the calculation would in principle yield the concentration of cuticular substances in ng per mm^2^ surface area for cockroaches or per mm^2^ squared head width for ants. However, the use of only a single non-hydrocarbon standard and the simplified calculation of the surface area only allow for rough estimates, so that we will only refer to “arbitrary units” to avoid the impression of an accurate measurement of concentration.

## Additional files

10.1186/s12898-016-0089-5 The cuticular substances of *Acromyrmex octospinosus* and *Atta colombica* workers and of *Attaphila* cockroaches collected from colonies of the two leaf-cutting ant species, with the relative abundances (mean and SD), retention times (rt), and principal component loadings (PC). Some substances were not found in one of the species (-). We also list the substance class as used for comparative statistics. “x-Me” refers to methylated alkenes with undetermined branch position.
10.1186/s12898-016-0089-5 The raw chemical data (total ion count) per sample for *Acromyrmex* colonies. Peaks are numbered according to Table 1 and additional file 1; sample names include information about colony number and whether the sample is a cockroach (“roach”) or a worker (“mw”).
10.1186/s12898-016-0089-5 The raw chemical data (total ion count) per sample for *Atta* colonies. Peaks are numbered according to Table 1 and additional file 1; sample names include information about colony identity (first word) and whether the sample is a cockroach (“roach”) or a worker (“mw”).
10.1186/s12898-016-0089-5 The data collected in the behavioural experiment. Included are raw numbers of the occurrences of different behaviours (ignorance, investigation, grooming, threat, biting) per replicate and information on whether (1) or not (0) a cockroach died within 48 h in the aggression arena.
