# Supplementary material for: Chemical disguise of myrmecophilous cockroaches and its implications for understanding nestmate recognition mechanisms in leaf-cutting ants
Source: BMC Ecol. 2016 Aug 5;16:35. doi: 10.1186/s12898-016-0089-5 (PMC4974750; doi:10.1186/s12898-016-0089-5)
Supplement: Supplementary file 1 — 10.1186/s12898-016-0089-5 The cuticular substances of Acromyrmex octospinosus and Atta colombica workers and of Attaphila cockroaches collected from colonies of the two leaf-cutting ant species, with the relative abundances (mean and SD), retention times (rt), and principal component loadings (PC). Some substances were not found in one of the species (-). We also list the substance class as used for comparative statistics. “x-Me” refers to methylated alkenes with undetermined branch position. [file 12898_2016_89_MOESM1_ESM.pdf]

Additional file 1: The cuticular substances of *Acromyrmex octospinosus* and *Atta colombica* workers and from *Attaphila* cockroaches collected from colonies of the two leaf-cutting ant species, with the relative abundances (mean and SD), retention times (rt), and principal component loadings (PC). Some substances where not found in one of the species, the respective cells are left blank. We also list the substance class as used for comparative statistics. "x-Me" refers to methylated alkenes with undetermined branch position.

				Samples from Acromyrmex colonies								Samples from Atta colonies											
				Worker		Cockroach						Worker		Cockroach									
No	Substance	Rt	Type	Mean	SD	Mean	SD	PC1	PC2	PC3	PC4	Mean	SD	Mean	SD	PC1	PC2	PC3	PC4	PC5	PC6	PC7	PC8
1	unidentified	5.3	other	0.34	0.23	0.70	0.73	-0.02	-0.13	-0.01	0.07	0.32	0.30	0.77	1.11	0.04	0.03	0.01	0.12	-0.12	0.11	-0.51	-0.07
2	C12:OH	6.7	other	2.42	1.46	2.86	2.00	0.28	0.03	0.00	-0.09	2.48	2.10	5.21	2.23	-0.21	0.18	0.04	0.12	0.11	-0.01	-0.21	-0.06
3	n-C18	11.2	linear	0.64	1.36	1.14	1.20	-0.09	0.07	0.13	-0.28	0.06	0.07	0.27	0.60	-0.12	-0.07	0.05	0.02	-0.27	-0.34	-0.21	0.00
4	C16-OH	12.6	other	0.21	0.21	0.30	0.28	-0.17	0.17	0.17	0.03	-	-	-	-	-	-	-	-	-	-	-	-
5	n-C20	14.9	linear	0.06	0.05	0.83	0.96	-0.07	0.11	0.27	-0.18	-	-	-	-	-	-	-	-	-	-	-	-
6	C18-OH	16.6	other	0.38	0.22	0.61	0.26	0.08	-0.02	0.16	0.06	0.27	0.40	0.35	0.27	0.02	-0.03	-0.30	0.13	-0.13	-0.06	-0.02	0.17
7	n-C22	19.0	linear	0.10	0.07	0.64	0.70	-0.05	0.13	0.25	-0.19	0.03	0.02	2.19	8.89	-0.12	-0.07	-0.14	0.19	-0.29	-0.06	-0.02	-0.11
8	n-C23	20.9	linear	0.31	0.24	0.29	0.29	-0.05	0.31	0.08	0.08	0.26	0.12	0.15	0.19	0.14	-0.27	0.09	-0.03	0.09	-0.22	0.10	-0.17
9	octadecenamide	22.1	other	0.70	0.53	0.46	0.49	-0.04	0.04	-0.04	0.26	0.44	0.61	0.32	0.42	-0.03	0.01	-0.21	-0.11	0.14	0.35	0.22	-0.17
10	n-C24	22.8	linear	0.24	0.17	0.77	0.79	-0.09	0.22	0.21	-0.14	0.26	0.16	0.26	0.50	0.08	-0.11	-0.09	0.16	-0.08	-0.38	0.06	0.11
11	n-C25	24.8	linear	1.19	0.79	0.55	0.53	0.01	0.29	-0.10	0.00	9.07	2.84	0.36	0.36	0.22	-0.27	0.01	-0.13	0.06	-0.09	0.04	-0.09
12	n-C26	26.6	linear	0.52	0.22	0.78	0.83	-0.03	0.29	0.02	-0.11	0.86	0.26	0.41	0.50	0.19	-0.28	-0.07	-0.05	0.03	-0.17	-0.06	-0.18
13	7-, 8-, 9-, 10-, 11-, 12-, 13-MeC26	27.2	methyl	1.01	0.55	1.06	0.48	0.25	-0.10	-0.11	-0.04	0.61	0.27	0.99	0.36	-0.10	0.21	-0.15	0.27	0.07	-0.13	-0.06	-0.13
14	n-C27	28.4	linear	4.55	2.40	0.59	0.44	0.02	0.22	-0.27	0.07	4.83	1.11	0.53	0.33	0.23	-0.18	0.00	-0.16	0.18	-0.10	0.00	0.01
15	7-, 9-, 11-, 13-MeC27	28.9	methyl	1.94	1.14	2.33	1.53	0.21	0.07	-0.07	-0.15	0.83	0.57	1.71	0.92	-0.22	0.15	0.07	-0.07	-0.11	-0.15	0.06	-0.27
16	docosenamide	29.7	other	13.97	14.65	15.57	11.07	0.20	-0.16	-0.11	-0.01	8.37	5.93	8.86	8.16	0.10	0.03	-0.28	0.02	-0.14	0.17	-0.15	-0.11
17	n-C28	30.0	linear	0.55	0.39	0.67	0.68	-0.10	0.28	0.00	-0.10	0.68	0.33	1.51	3.83	0.06	0.01	-0.08	-0.24	0.13	-0.11	-0.32	0.13
18	9-, 10-, 11-, 12-, 13-, 14-MeC28	30.6	methyl	2.87	4.27	3.47	6.17	0.19	0.04	0.05	0.07	1.07	0.71	1.95	1.21	-0.20	0.04	0.28	0.06	0.12	-0.18	0.09	-0.07
19	C29:2a	30.7	unsat	0.45	0.68	0.19	0.19	-0.09	0.10	-0.21	0.04	-	-	-	-	-	-	-	-	-	-	-	-
20	C29:2b	30.8	unsat	0.47	0.62	0.27	0.24	-0.13	0.05	-0.08	0.05	-	-	-	-	-	-	-	-	-	-	-	-
21	C29:1	31.4	unsat	10.90	12.01	2.60	2.31	-0.08	-0.05	-0.25	-0.07	-	-	-	-	-	-	-	-	-	-	-	-
22	n-C29	31.8	linear	3.06	1.92	5.61	1.82	-0.11	-0.16	0.12	-0.05	9.93	3.83	12.19	6.00	0.05	0.30	-0.16	-0.12	0.18	-0.03	-0.12	0.08
23	9-, 11-, 13-MeC29	32.2	methyl	3.88	2.53	4.64	2.21	0.19	0.06	0.00	-0.17	1.41	0.88	3.05	2.38	-0.27	0.10	0.06	0.03	0.12	-0.07	0.15	-0.15
24	3-MeC29 + methylated alkanes	32.9	methyl	1.32	1.22	0.95	0.69	0.12	0.20	0.11	0.16	3.09	3.95	2.18	2.99	-0.12	-0.14	-0.13	0.01	0.04	0.10	0.21	0.40
25	10-, 11-, 12-, 13-, 14-, 15-MeC30	33.8	methyl	3.60	2.87	2.35	1.30	0.28	0.05	-0.02	-0.02	2.19	1.47	3.91	2.61	-0.19	0.03	0.06	0.03	0.20	-0.06	0.02	-0.17

26	C31:2	34.1	unsat	5.88	9.33	5.94	3.61	-0.21	-0.13	0.08	-0.12	-	-	-	-	-	-	-	-	-	-	-	-
27	C31:1	34.6	unsat	3.71	3.81	5.65	2.50	-0.20	-0.20	0.06	-0.02	-	-	-	-	-	-	-	-	-	-	-	-
28	N-C31 and 2Me-C30	34.9	linear	1.31	0.87	4.06	2.88	-0.05	-0.21	0.13	0.04	0.81	0.49	1.49	0.81	-0.12	0.01	0.31	-0.01	0.04	-0.07	-0.19	0.26
29	9-, 11-, 13-, 15-Me-C31	35.4	methyl	4.26	2.44	5.18	1.80	0.22	-0.08	-0.12	-0.25	1.44	1.04	2.52	1.61	-0.17	-0.01	0.07	0.08	0.17	0.07	-0.17	-0.22
30	7,11-diMe-C31	36.0	methyl	-	-	-	-	-	-	-	-	0.55	0.81	0.84	0.86	-0.01	0.09	0.09	0.14	0.35	0.03	0.03	-0.01
31	10-, 11-, 12-, 13-, 14-, 15-, 16-MeC32	37.2	methyl	1.62	1.86	2.19	0.97	0.24	-0.10	0.05	-0.04	-	-	-	-	-	-	-	-	-	-	-	-
32	3,7,11-triMe-C31	37.3	methyl	-	-	-	-	-	-	-	-	11.91	3.17	7.95	3.44	0.22	0.18	0.02	-0.17	0.18	0.01	-0.03	0.02
33	C33:2	37.4	unsat	4.02	4.49	6.83	3.37	-0.22	-0.12	-0.04	-0.22	0.22	0.09	0.22	0.16	0.18	0.21	0.00	-0.07	0.20	-0.03	-0.01	0.12
34	8,12-diMe-C32	37.7	methyle	0.84	0.90	1.33	1.31	-0.04	-0.07	0.00	-0.15	-	-	-	-	-	-	-	-	-	-	-	-
35	C33:2	37.7	unsat	0.84	0.90	1.33	1.31	-0.04	-0.07	0.00	-0.15	0.98	2.86	0.69	2.37	-0.13	0.01	0.04	-0.05	-0.15	0.06	0.31	0.25
36	6,10-diMe-C32	37.9	methyl	-	-	-	-	-	-	-	-	0.15	0.13	0.33	0.25	-0.23	0.01	-0.11	-0.24	0.16	0.03	0.00	0.03
37	n-C33 + methylated alkane	38.2	linear	0.71	0.52	0.93	0.43	-0.06	-0.10	0.15	0.26	0.27	0.22	0.84	0.61	-0.21	-0.02	-0.11	-0.26	0.11	0.05	-0.01	-0.01
38	9-, 11-, 13-, 15-MeC33	38.8	methyl	2.04	1.73	2.80	1.21	0.15	-0.15	-0.08	-0.28	-	-	-	-	-	-	-	-	-	-	-	-
39	9,13-diMe-C33	39.5	methyl	0.59	0.69	0.14	0.11	0.14	0.05	-0.20	-0.05	0.35	0.68	0.18	0.11	0.13	0.01	-0.05	0.24	0.19	-0.15	-0.05	-0.09
40	3Me-C33	40.0	methyl	1.49	1.43	1.27	1.51	0.19	0.18	0.12	0.08	0.37	0.31	1.28	1.77	-0.17	-0.05	0.05	0.20	-0.14	0.12	0.13	0.05
41	C35:1 + C35:2	41.0	unsat	3.33	4.31	5.31	4.43	-0.07	-0.08	0.25	0.04	-	-	-	-	-	-	-	-	-	-	-	-
42	methyle alkane mixture	42.0	methyl	-	-	-	-	-	-	-	-	4.74	1.63	4.69	1.52	0.19	0.27	0.00	0.08	-0.03	0.02	0.01	0.00
43	4,8,12-triMe-C34	42.6	methyl	-	-	-	-	-	-	-	-	12.60	4.34	14.60	5.91	0.05	0.10	0.37	-0.11	-0.19	-0.05	-0.06	0.18
44	13-, 15-, 17-MeC35	42.6	methyl	0.64	0.78	1.11	0.40	0.06	-0.20	0.13	-0.17	-	-	-	-	-	-	-	-	-	-	-	-
45	x-MeC36:1	42.9	unsat	-	-	-	-	-	-	-	-	0.40	0.47	0.35	0.75	0.11	-0.02	0.27	0.01	0.02	0.20	-0.17	0.18
46	x-MeC36:1	43.1	unsat	-	-	-	-	-	-	-	-	0.31	0.25	0.91	3.06	0.06	-0.05	0.19	0.17	-0.05	0.31	0.02	0.03
47	x-MeC36:1	43.3	unsat	-	-	-	-	-	-	-	-	0.46	0.28	0.32	0.21	0.12	-0.09	0.35	-0.05	-0.06	0.09	0.10	-0.21
48	5,9- and 5,11-diMe-C35	43.9	methyl	-	-	-	-	-	-	-	-	3.75	1.21	3.32	1.09	0.16	0.03	0.09	0.10	-0.14	0.24	-0.03	-0.17
49	3,7,11-triMe-C35	45.0	methyl	-	-	-	-	-	-	-	-	5.05	6.62	3.81	2.00	0.13	0.11	0.07	0.13	-0.09	0.11	0.04	0.05
50	C37:2 + C37:1	45.0	unsat	5.59	6.78	3.50	2.07	0.06	-0.13	0.22	0.30	-	-	-	-	-	-	-	-	-	-	-	-
51	x-MeC37:1	45.3	unsat	-	-	-	-	-	-	-	-	0.55	0.72	0.65	0.83	0.07	0.19	-0.09	0.05	-0.05	-0.22	0.20	0.17
52	4,8,12-triMe-C36	46.6	methyl	-	-	-	-	-	-	-	-	2.61	0.79	2.65	0.77	0.18	0.30	-0.05	0.14	-0.06	-0.10	0.06	0.05
53	x-MeC38:1	47.0	unsat	0.75	0.42	0.64	0.54	0.12	0.24	0.04	0.18	1.07	0.72	0.93	0.70	-0.06	-0.24	-0.17	0.06	-0.03	0.20	-0.14	-0.03
54	x-MeC40:1	48.5	unsat	-	-	-	-	-	-	-	-	2.16	1.23	2.47	1.22	0.02	0.18	-0.08	-0.29	-0.28	0.00	0.06	-0.27
55	x-MeC40:1	49.2	unsat	4.44	5.83	1.96	1.25	0.17	-0.11	0.15	0.27	0.66	0.30	0.59	0.27	0.06	0.19	0.09	-0.35	-0.25	-0.05	0.10	-0.16
56	x-MeC41:1	50.1	unsat	-	-	-	-	-	-	-	-	1.13	0.43	0.99	0.35	0.22	0.23	-0.01	0.11	-0.02	0.03	0.14	-0.07
57	x-MeC41:1	51.4	unsat	-	-	-	-	-	-	-	-	0.37	0.47	0.22	0.11	0.16	-0.07	0.06	0.28	0.14	-0.03	0.22	-0.15

58	x-MeC41:2	53.8	unsat	0.72	1.03	0.21	0.20	0.13	-0.13	-0.01	0.23	-	-	-	-	-	-	-	-	-	-	-	-
59	13-, 15-Me-C41	55.1	methyl	0.38	0.47	0.13	0.10	-0.15	-0.01	-0.23	0.12	-	-	-	-	-	-	-	-	-	-	-	-
60	alkyl ester 1	58.8	other	0.55	0.70	0.20	0.29	-0.14	-0.02	-0.25	0.05	-	-	-	-	-	-	-	-	-	-	-	-
61	x-MeC44:1	59.2	unsat	0.53	0.50	0.30	0.19	-0.21	-0.05	-0.14	0.09	-	-	-	-	-	-	-	-	-	-	-	-
62	alkyl ester 2	63.9	other	0.90	0.83	0.15	0.09	-0.12	0.02	-0.28	0.10	-	-	-	-	-	-	-	-	-	-	-	-
